# Hearing Benefit in Allograft Tympanoplasty Using Tutoplast Processed Malleus

**DOI:** 10.1155/2014/931308

**Published:** 2014-02-13

**Authors:** Anja Lieder, Wolfgang Issing

**Affiliations:** Department of Otolaryngology, Freeman Hospital, Freeman Road, High Heaton, Newcastle upon Tyne NE7 7DN, UK

## Abstract

*Objectives.* Tutoplast processed human cadaveric ossicular allografts are a safe alternative for ossicular reconstruction where there is insufficient material suitable for autograft ossiculoplasty. We present a series of 7 consecutive cases showing excellent air-bone gap closure following canal-wall-down mastoidectomy for cholesteatoma and reconstruction of the middle ear using Tutoplast processed malleus. *Patients and Methods.* Tympanoplasty with Tutoplast processed malleus was performed in seven patients to reconstruct the middle ear following canal-wall-down mastoidectomy in a tertiary ENT centre. *Main Outcome Measures.* Hearing improvement and recurrence-free period were assessed. Pre-and postoperative audiograms were performed. *Results.* The average pre operative hearing loss was 50 ± 13 dB, with an air-bone gap of 33 ± 7 dB. Post operative audiograms at 25 months demonstrated hearing thresholds of 29 ± 10 dB, with an air-bone gap of 14 ± 6 dB. No prosthesis extrusion was observed, which compares favourably to other commercially available prostheses. *Conclusions.* Tutoplast processed allografts restore conductive hearing loss in patients undergoing mastoidectomy and provide an excellent alternative when there is insufficient material suitable for autograft ossiculoplasty.

## 1. Introduction

Human cadaveric allografts have been used in middle ear reconstruction for half a century. They fully integrate into the middle ear and may be used to reconstruct the ossicular chain where there is insufficient autologous material. Tutoplast processed ossicular allografts (Tutoplast Ossicula auditus) consist of dehydrated human malleus or incus and provide a matrix for new bone formation through bone remodelling. They are derived from selected donors using the Tutoplast process. This process involves osmotic destruction of tissue cells, followed by denaturation using sodium hydroxide and hydrogen peroxide to inactivate all pathogens, and finally dehydration and sterilization by gamma irradiation. The Tutoplast process inactivates all living organisms and spores from donated tissue and achieves Sterility Assurance Level of 10^−6^. Each transplant can be tracked back to the original donor [1, 2]. Tutoplast Ossicula auditus is licensed as a medical product in Germany and fulfils European Union and USA medical drug regulations.

We present a series of 7 consecutive cases demonstrating excellent long-term hearing improvements in tympanoplasty using Tutoplast processed malleus to reconstruct the middle ear following mastoidectomy.

## 2. Patients and Methods

Seven consecutive patients with cholesteatoma aged 11–69 years (four male, three female) underwent canal-wall-down mastoidectomy and tympanoplasty between May 2009 and January 2011. Two cases were revisions. All patients had canal-wall-down mastoidectomy for removal of cholesteatoma, followed by ype III tympanoplasty including myringoplasty with tragal perichondrium in a single-stage procedure. This would entail the placement of a Tutoplast processed malleus (Tutoplast Ossicula auditus, Tutogen Medical GmbH, Neunkirchen, Germany) onto the stapes if the patient's own ossicles were found to be either absent, eroded or unsuitable for an autograft. One patient had additional reconstruction of posterior canal wall using tragal cartilage. All patients had their ear dressed with 2 silastic sheets, one being placed in the mastoid cavity to facilitate epithelialisation of the cavity and the other to cover the tympanic membrane. Bismuth iodine paste gauze dressing was applied into the external auditory meatus for 2-3 weeks. Once sufficient healing was ascertained, patients were instructed in the Valsalva manoeuvre and were encouraged to perform it 20–30 times per day.

Hearing assessment was by pure tone audiograms in accordance with the British Society of Audiology recommended procedure (2004). Values are given in Decibel Hearing Level (dB HL) for testing frequencies of 250, 500, 1000, 2000, 4000, and 8000 Hertz (Hz). Air-bone gaps were calculated in accordance with the American Academy of Otolaryngology-Head and Neck Surgery (AAO-HNS) 1995 guideline. The testing frequency of 3000 Hz was substituted with 4000 Hz. Audiograms were read by two observers independently. Average hearing levels are given in dB HL and standard deviations are applied where appropriate. Where applicable, Student's *t*-test (equal sample size, unequal variance) was performed and *P* values were given.

## 3. Ethical Considerations

Written consent, including the use of Tutoplast Ossicula auditus, was obtained. All investigations and procedures were performed according to best clinical practice and the medical principles of the Declaration of Helsinki. The National Research Ethics Service of the United Kingdom has confirmed that formal ethics approval procedure is not required (NRES Ref 04/26/31) as this is a retrospective study using a fully licensed product.

## 4. Results

Postoperative complications were not observed. The stapes suprastructure was fully intact in six patients. There was partial destruction of the stapes suprastructure in one patient, with one crus being present, and, in this case, the prosthesis was placed on the preserved crus.

The average preoperative hearing loss (air conduction) was 49.5 ± 12.7 dB (36–73 dB). The average preoperative air-bone gap was 32.7 ± 6.6 dB (23–43 dB) (Table 1). Patients were followed up between 15 and 34 months after surgery, on average 25 ± 6 months. Recurrence or prosthesis extrusion was not observed. All patients had a safe dry ear upon clinical examination and reported substantial improvement to their hearing.

**Figure 1:**
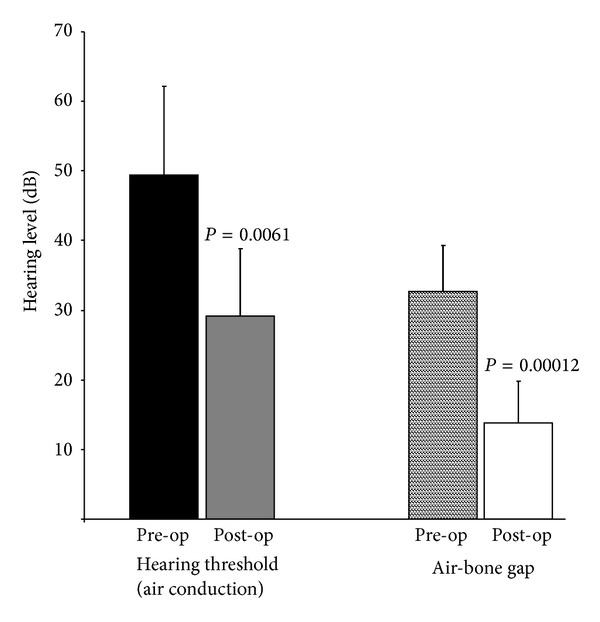
Hearing thresholds before and after surgery. Preoperative (pre-op) and postoperative (post-op) hearing thresholds in db Hearing Level including standard deviation are shown. The first group of two columns (black and grey column, resp.) denotes the hearing threshold on air conduction preoperatively and postoperatively. The second group of two columns denotes the air-bone gap preoperatively and postoperatively (patterned and white column, resp.).

**Figure 2:**
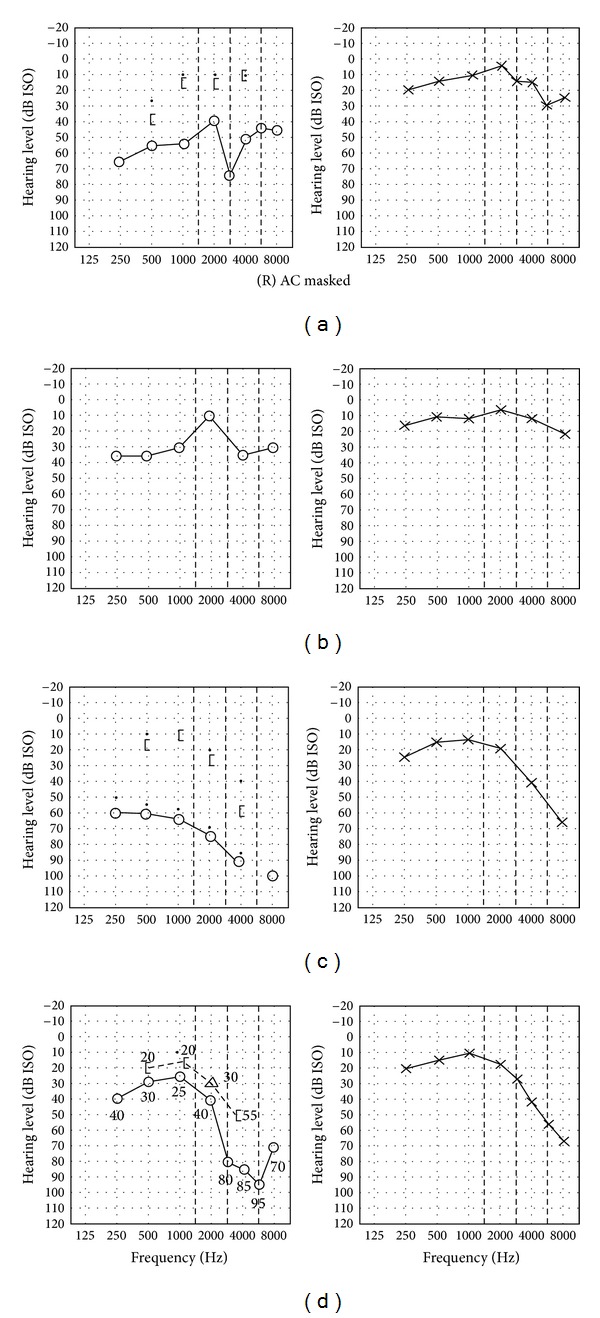
Representative pre- and postoperative original PTA from two patients. Two representative preoperative and postoperative PTA of two patients. Patient 1 (rows (a) and (b)) showed good closure of his operated right ear ABG from 30 dB to 18 dB postoperatively. Patient 4 (rows (c) and (d)) showed excellent ABG closure over 29 dB with a residual ABG of 14 dB in the operated right ear.

Postoperative hearing thresholds (air conduction) in the operated ear had improved to 29.1 ± 9.7 dB (*P* = 0.006). The air-bone gap had narrowed to 13.8 ± 6.0 dB after surgery (4–24 dB) (*P* = 0.0001). Six patients (86%) had a postoperative air-bone gap of less than 20 dB. The air-bone gap closure achieved was on average 18.9 ± 8.5 dB (4–29 dB, Table 1 and Figure 1). Representative pre- and postoperative pure tone audiograms are shown in Figure 2.

## 5. Discussion

Middle ear reconstruction following cholesteatoma surgery can be challenging. Auditory ossicles are often eroded, making them insufficient for ossiculoplasty, and they can also harbour remnants of cholesteatoma matrix which can facilitate disease recurrence. Surgical options in these cases include the use of ossicular replacement prostheses or ossicular allografts. Apprehension in using such allografts over a fear of infection transmission has not made them widely known surgical options in recent years and many surgeons have no experience in using them. Tutoplast processed malleus is safe. Since the inception of Tutoplast processed human cadaveric allografts in the 1970s and, there have been no reported cases of graft rejection or disease transmission [2].

Tutoplast processed malleus acts as a collagen matrix for bone regeneration and remodelling. Studies on allograft ossiculoplasty dating back to the 1970s demonstrate that allograft ossicles (notched incus homograft) achieve excellent integration and restoration of hearing [3]. Tutoplast processed bone grafts achieve the highest mesenchymal stem cell adherence in vitro, hence making it an ideal environment for bone regeneration [4]. A study on a postmortem temporal bone confirms minimal resorption of allograft ossicles [5] and longevity of these grafts is excellent, as no osteoclastic bone resorption occurs [6].

Ossicular replacement prostheses are used in middle ear reconstruction with extensively published evidence. A series of 465 cases reported closure of air-bone gap to ≤ 15 dB in 63% of cases and to ≤ 20 dB in 73% of cases with partial ossicular replacement prostheses (PORP) [7]. A series of 650 cases, also using Plastipore PORP, reported postoperative air-bone gaps of ≤ 20 dB in 68%, although the average air-bone gap was 18 ± 11 dB after 12 months [8]. Another group reported postoperative air-bone gap closure (≤20 dB) in tympanoplasty following mastoidectomy for 46% and 33% for titanium and hydroxyapatite prostheses, respectively. Average postoperative air-bone gap was 26.5 dB, with 23.8 dB for titanium group and 29.8 dB for hydroxyapatite group after 1 year [9]. Closure of the air-bone gap fourteen years following mastoidectomy and tympanoplasty using Plastipore PORP was reported to be 60% in a group of 5 patients [10].

Tympanoplasty with allogeneic ossicles can restore hearing to levels comparable to autograft, and hearing benefit is often favourable to prostheses. Early reports by Wehrs report a graft take rate between 92 and 96% and a satisfactory hearing outcome between 77 and 89% [11]. In a case series on using homologous or autologous incus interposition grafts, there was no significant difference in hearing gain between allografts and autografts. Postoperative air-bone gap was 19 dB, with 66% of patients achieving an air-bone gap closure of 20 dB or better after 15 months [12]. Another study on malleus allograft ossiculoplasties reported air-bone gap closure of ≤ 20 dB in 81% of cases one year postoperatively, but, in all cases, stapes suprastructure was missing and ossiculoplasty was performed as a secondary procedure, making these outcomes less straightforward to compare [13].

Others report less favourable hearing outcomes compared to autografts or glass ionomer cement. In a study of 293 patients comparing different means of ossicular reconstruction, cholesteatoma removal was the primary cause for surgery in 62 cases (21%), with a mean postoperative air-bone gap of 15 ± 8 dB. Allograft ossicles were used in 39 out of 293 cases (13%), resulting in a postoperative air-bone gap of 13 ± 9 dB (mean air-bone gap closure 17 ± 9 dB). There is no distinct group undergoing canal-wall-down mastoidectomy for cholesteatoma using allograft ossicles in this study, which again makes this difficult to compare with other studies [14].

Our postoperative air-bone gap is 13.8 dB. The average closure of the air-bone gap is 18.9 dB, and 86% of patients had a postoperative air-bone gap of 20 dB or less. Our outcomes gained from a single-stage procedure exceed the air-bone gap closures reported for PORP in some of the larger studies of tympanoplasty [7, 8]. They also compare favourably to results achieved in canal-wall-down mastoidectomy [9, 10, 13]. Moreover, the majority of operations in these studies were performed for chronic suppurative otitis media or were secondary procedures, and canal-wall-down mastoidectomy was either performed as a separate stage or not at all.

In addition to potentially advantageous hearing benefit, allogeneic ossicular grafts integrate into the middle ear and rarely extrude, with historic failure rates between 4 and 8% [11] and more recent extrusion rates of 0% [13]. Extrusion rates between 4-5% [8, 10] and 7% [7] have been reported in studies using prostheses. No extrusion was observed in our series but, due to its low group size, a conclusion on graft extrusion rates is not possible.

## 6. Conclusion

- We demonstrate that Tutoplast processed malleus restores hearing in 7 patients undergoing canal-wall-down mastoidectomy and tympanoplasty for cholesteatoma.
- We recommend consideration of Tutoplast processed malleus in cases of cholesteatoma where autologous material cannot be used and where a single-step operative procedure to eradicate cholesteatoma with concomitant reconstruction of the middle ear is desired.
- In this small study with a 25-month follow-up, no graft extrusion or other complications were observed, and we are encouraged to offer allograft ossicular reconstruction as an alternative to ossicular prostheses to our patients undergoing mastoid exploration for cholesteatoma who wish to have ossiculoplasty but who have insufficient autologous ossicles.

## Figures and Tables

**Table 1:** Comparison between pre- and postoperative audiograms and assessment of air-bone gap.

	Patient								
	1	2	3	4	5	6	7	Mean	SD
Operated ear									
Preoperative hearing (air conduction; 500–4000 Hz)	50	41	55	73	54	38	36	**49.5**	*12.7 *
Preoperative air-bone gap (500–4000 Hz)	30	23	28	43	38	34	35	**32.7**	*6.6 *
Follow-up (months)	34	26	29	29	20	22	15	25	*6 *
Postoperative hearing (air conduction; 500–4000 Hz)	28	28	39	45	28	19	19	**29.1** ^ a^	*9.7 *
Postoperative air-bone gap (500–4000 Hz)	18	5	24	14	15	9	13	**13.8** ^ b^	*6.0 *
Air-bone gap closure achieved	13	18	4	29	23	25	23	18.9	*8.5 *

^a^ *P* = 0.0061; ^b^ *P* = 0.00012. Bold: Mean values. Italic: Standard deviation.
